# Role of miRNA signalling in the pathogenesis of MASLD

**DOI:** 10.3389/fphar.2026.1756805

**Published:** 2026-04-15

**Authors:** Shi-Lin Cheng, Jun Hao, Hong-Yan Qin

**Affiliations:** 1 State Key Laboratory of Holistic Integrative Management of Gastrointestinal Cancers, Department of Medical Genetics and Developmental Biology, Fourth Military Medical University, Xi’an, China; 2 The College of Life Sciences, Northwest University, Xi’an, China

**Keywords:** inter-organ communication, MASH, MASLD, miRNA, therapeutics

## Abstract

MicroRNAs (miRNAs) are short non-coding RNA molecules that are involved in metabolic dysfunction-associated steatotic liver disease (MASLD) either by promoting or inhibiting the transition from hepatic steatosis to steatohepatitis/fibrosis or even hepatocellular carcinoma. Mechanistically, miRNAs regulate the progression of MASLD through intercellular communication within hepatic tissues or remote transport into the liver from extrahepatic tissue. In comparison with conventional RNA-based therapeutics, miRNAs show irreplaceable advantages for MASLD treatment, including reduced immunogenicity, enhanced therapeutic precision, and improved structural stability. This review systematically summarizes the assignable roles of miRNA-mediated intrahepatic or extrahepatic communication in MASLD, and evaluates the existing miRNA-targeting therapeutic strategies for MASLD.

## 1 Introduction

Metabolic dysfunction-associated steatotic liver disease (MASLD), previously termed non-alcoholic fatty liver disease (NAFLD), is now recognized to present not only with hepatic lipid accumulation, but also with systemic metabolic disorders such as obesity, hypertriglyceridemia, hypertension, and hyperglycemia (Tak et al., 2025). MASLD has become the leading cause of chronic liver disease, affecting more than 30% of the global population, and represents a serious health burden for humanity (Zeng X. L. et al., 2024). MASLD can progress from simple steatosis to metabolic dysfunction-associated steatohepatitis (MASH), which may be accompanied by liver fibrosis, cirrhosis, and even liver cancer. Steatosis, the primary feature of MASLD, is defined as the accumulation of lipids in more than 5% of hepatic parenchymal cells in the absence of excessive alcohol consumption, drug use, or other predisposing factors (Esparza et al., 2023; Konings et al., 2025). During steatosis, *de novo* lipogenesis (DNL) in the liver maintains metabolic homeostasis, while excess levels of triglycerides inhibit the secretion of very low-density lipoprotein (vLDL) through negative feedback. The accumulation of liver lipids accelerates the inflammatory process, causing oxidative stress and disruption of cellular structures, including cell membranes, endoplasmic reticulum, and mitochondria, and ultimately resulting in the transition from metabolic dysfunction-associated fatty liver (MAFL) to MASH (Tauil et al., 2024). MASH may occur with or without liver fibrosis, and its pathological manifestations include steatosis, hepatocyte ballooning, lobular inflammation, portal granulocytic inflammation, and Mallory–Denk bodies (Neuman et al., 2015; Tsuchida et al., 2018; Shan et al., 2019). MASH is also considered a major driving factor for cirrhosis, with approximately 20%–50% of patients progressing to cirrhosis within 10 years, and up to 40% of these cases subsequently developing liver cancer (Kong et al., 2020). Furthermore, MASLD not only affects the liver but can also cause extrahepatic complications, including cardiovascular disease (CVD), osteoporosis, chronic kidney disease, and depression (Zeng Z. L. et al., 2024). Due to the complexity of the presentation of MASLD, highly effective strategies for treating this condition are lacking at present.

The existing therapeutic options for MASLD primarily rely on lifestyle modifications, including dietary changes, exercise, and behavioral interventions. However, these strategies are often hindered by poor adherence and limited effectiveness in reversing advanced disease, necessitating pharmacological or surgical adjuvant combination therapies (Alabdul Razzak et al., 2024; Wan et al., 2025). Traditional therapeutic agents for MASLD include peroxisome proliferator-activated receptor (PPAR)-γ agonists, glucagon-like peptide (GLP)-1 analogs, sodium-dependent glucose transporter (SGLT)-2 inhibitors, fibroblast growth factor 21 (FGF21) analogs (e.g., pegbelfermin), and farnesoid X receptor (FXR) modulators, while FDA-approved new drugs such as resmetirom target metabolic regulation, anti-inflammatory effects, or anti-fibrotic mechanisms (Stojsavljevic-Shapeski et al., 2020; Harrison et al., 2024). In contrast, emerging therapies involving RNA-based drugs have demonstrated substantial potential for MASLD treatment. The most prevalent types of RNA drugs include small interfering RNA (siRNA), messenger RNA (mRNA), antisense oligonucleotides (ASOs), and microRNA (miRNA). These RNA therapeutics function by modulating the expression of targeted molecules involved in the progression of MASLD (La Sala et al., 2024). In this regard, miRNAs show remarkable advantages over other RNA drugs in terms of immunogenicity, treatment precision, and stability.

miRNAs are endogenous non-coding RNAs with a length of approximately 22 nucleotides. Extracellular miRNAs are predominantly packaged within exosomes, a major type of extracellular vesicles, which include exosomes (30–120 nm), microvesicles (50–1,000 nm), and apoptotic bodies (usually >500 nm) (Qi and Lai, 2022). During the miRNA sorting process, RNA-binding proteins (RBPs) recognize specific sequences or structural motifs (EXO motifs) on miRNAs, which guide the loading of miRNAs into exosomes or multivesicular bodies (MVBs) for transport (Akuta et al., 2019; Wu et al., 2025). miRNAs play a crucial role in post-transcriptional regulation by targeting mRNA, which can lead to mRNA degradation and inhibition of protein translation (Ha and Kim, 2014; Condrat et al., 2020). Mechanistically, the miRNA-induced silencing complex (miRISC) binds to the 3′-untranslated region (UTR) of target mRNAs, recruiting the chromatin assembly factor-1 (CAF1)-carbon catabolite repression 4 (CCR4)-negative on TATA-less (NOT) deadenylase complex along with poly(A)-binding proteins (PABPs) and anti-proliferative (APRO) family proteins to promote mRNA deadenylation, degradation, or translational repression. Notably, N^6^-methyladenosine (m^6^A) modification synergistically enhances miRNA-mediated regulation. The m^6^A reader protein YTHDF2 interacts with the miRISC and recruits the CCR4-NOT complex to promote the decay of m^6^A-modified mRNAs. Conversely, miRNAs can modulate the expression of m^6^A modification enzymes, thereby indirectly influencing the m^6^A methylation landscape of target transcripts. In contrast, circular RNAs (circRNAs) function as miRNA sponges by sequestering miRNAs through complementary binding sites, thereby preventing miRNA-mRNA interactions. Acting as competitive endogenous RNAs (ceRNAs), circRNAs alleviate miRNA-mediated repression and restore the expression of otherwise silenced target genes (Nakashima et al., 2024). In the context of MASLD, miRNAs are crucial for regulating lipid metabolism, glucose homeostasis, cell proliferation, apoptosis, migration, and differentiation (Carpi et al., 2024). In RNA-based technology, engineered miRNAs are emerging as potential therapeutic agents and drug targets. However, a comprehensive understanding of the targets and regulatory mechanisms of miRNAs is essential to develop miRNA-based therapies. Therefore, this review aimed to elucidate the most recent advancements in the regulatory mechanisms underlying miRNA-mediated intrahepatic and extrahepatic communication during MASLD progression, and thereby provide novel insights and methodologies for diagnosis and treatment of human disease.

## 2 Overview of MASLD

### 2.1 Intrahepatic effects of MASLD

The pathogenesis of MASLD involves interactions among various intrahepatic cells, such as hepatocytes, Kupffer cells (KCs), T cells, natural killer (NK) cells, hepatic satellite cells (HSCs), and hepatic sinusoid epithelial cells (LSECs). Cytokines and miRNAs, as cell-cell mediators, play very important roles in driving the progression of MASLD.

#### 2.1.1 Cytokine-mediated crosstalk among intrahepatic cells in MASLD

Hepatocytes, the primary metabolic cell population in the liver, can initiate immune cell inflammatory responses by producing certain acute-phase proteins during liver injury (Yahoo et al., 2023). For instance, at the stage of hepatic steatosis, hepatocytes release interleukin (IL)-8, IL-1β, cellular communication network factor 1 (CCN1), and tumor necrosis factor (TNF)-α, which can exacerbate hepatic inflammation (Peiseler et al., 2022). Additionally, lipid-laden hepatocytes release damage-associated molecular patterns (DAMPs) that are recognized by pattern recognition receptors (PRRs) expressed on innate immune cells, further amplifying inflammatory responses. Concurrently, gut-derived lipopolysaccharide (LPS) translocates across the intestinal epithelium and binds to Toll-like receptor 4 (TLR4), activating the nuclear factor kappa B (NF-κB) signaling pathway that promotes the production of cytokines and proliferation of macrophages (Softic et al., 2019). In addition, senescent hepatocytes also release inflammatory signals. Innate immune cells, including macrophages, neutrophils, and NK cells, as well as adaptive immune cells such as CD4^+^ T, CD8^+^ T cells, and Th17 cells also participate in the progression of MASLD, contributing to liver inflammation, fibrosis, and hepatocellular carcinoma (HCC).

Macrophages, as the most abundant and heterogeneous cell population in the liver, play a crucial role in the progression of MASLD. At the early stages of MASLD, tissue-resident macrophage KCs (ResKCs) help maintain tissue homeostasis by clearing apoptotic cells and lipids, predominantly exhibiting an anti-inflammatory M2-like phenotype. In the MASH and fibrosis stages, circulating monocytes (Ly6C^+^ CCR2^+^) are recruited to the liver, where they differentiate into monocyte-derived macrophages (MoMFs) to amplify the inflammatory response and promote fibrosis. Notably, as the disease progresses, the number of ResKCs gradually decreases, while the number of CLEC4F^−^MoKCs increases. RNA sequencing (RNA-seq) analyses showed that these MoKCs are similar to lipid-associated macrophages (LAMs) in adipose tissue and scar-associated macrophages (SAMs) in the fibrotic human liver, and are therefore referred to as hepatic LAMs (Remmerie et al., 2020). However, hepatic LAMs and ResKCs and MoKCs also show differences in terms of their activation state and lipid metabolism ability. In addition, although TREM2^+^CD9^+^ SAMs and hepatic LAMs show overlapping gene expression profiles, SAMs represent a terminally differentiated cell state within the fibrotic niche and show more prominent expression of secreted phosphoprotein 1 (SPP1), whereas hepatic LAMs exhibit higher expression levels of genes related to lipid metabolism (Jaitin et al., 2019; Ramachandran et al., 2019; Guilliams et al., 2022). Under lipotoxic stimulation, KCs recruit other immune cells and infiltrated monocytes, inducing apoptosis and phagocytosis of damaged hepatocytes, followed by secretion of TNF-α, IL-1β, and transforming growth factor (TGF)-β to activate HSCs and induce fibrosis. In turn, ligands on HSCs, such as (C-C motif) ligand 2 (CCL2), regulate the activity of other immune cells and macrophages to aggravate fibrosis (Tran et al., 2020; Wang and Gao, 2020; Alabdulaali et al., 2023). Except cytokines, some key signaling pathways, such as the TLR4/NF-κB pathway, NOD-like receptor protein (NLRP3) inflammasomes, and metabolic reprogramming, also regulate macrophage activation. LPS and free fatty acid (FFAs) serve as activators of TLR4, and TLR4 activation further exacerbates insulin resistance and chronic inflammation. NF-κB enhances the transcriptional expression of pro-IL-1β and NLRP3, while NLRP3 inflammasome activation triggers the activation and cleavage of caspase 1, promoting proteolytic processing and secretion of mature IL-1β (Wayal et al., 2025). A growing body of evidence suggests that metabolic reprogramming exerts a very important role in macrophage activation, with pro-inflammatory M1 macrophages relying on glycolysis while anti-inflammatory M2 macrophages depend on oxidative phosphorylation (Horn and Tacke, 2024; Miao et al., 2024).

In addition to macrophages, other immune cells also play important roles in maintaining liver homeostasis. Notably, CD8^+^ T cells play complex roles in the progression of MASLD. On one hand, CXCR6^+^ CD8^+^ T cells, which are enriched in both mice with MASH and human patients, exhibit characteristics of forkhead box O1 (FOXO1) downregulation and C-X-C motif chemokine receptor 6 (CXCR6) upregulation upon IL-15 induction, rendering them sensitive to metabolic stimuli such as acetate and extracellular ATP. This sensitivity triggers a form of non-specific killing referred to as “auto-attack.” The process involves rapid upregulation of Fas ligand (FasL) following ATP exposure, and blockade of FasL has been shown to inhibit this auto-aggressive behavior *in vitro* and *in vivo* and thereby ameliorate liver injury (Dudek et al., 2021). On the other hand, liver-resident memory CD8^+^ T cells induce apoptosis of HSCs in a C-C motif chemokine receptor 5 (CCR5)-dependent manner through the FasL-Fas signaling pathway. Meanwhile, IL-15, acting through the IL-12RB receptor on these cells, delays the resolution of fibrosis (Koda et al., 2021). Furthermore, studies have indicated that CD8^+^ PD1^+^ T cells contribute to the development of MASH-HCC (Pfister et al., 2021). Obesity-induced lipid accumulation is associated with the loss of CD4^+^ T cells, and depletion of CD4^+^ T cells exacerbates the progression of HCC (Ma et al., 2016; Weiskirchen and Tacke, 2016). Interestingly, adoptive transfer of CD4^+^ T cells converted double-negative T cells (cDNTs) by protecting mice from diet-induced liver fat accumulation, lobular inflammation, and focal necrosis. DNTs, which are characterized as TCRαβ^+^CD3^+^CD4^−^CD8^−^NK1.1^−^ cells in mice and TCRαβ^+^CD3^+^CD4^−^CD8^−^CD56^−^ cells in humans, selectively suppress liver-infiltrating Th17 cells and pro-inflammatory M1 macrophages. IL-10 secreted by M2 macrophages reduces the survival and function of cDNTs, thereby protecting M2 macrophages from cDNT-mediated lysis (Sun et al., 2021). Th17 can promote the progression of NAFL to MASH through the release of IL-17 and may also drive hepatic fibrosis. Specific knockout of Hivep1 in IL-17A^+^CD4^+^ T cells has been shown to impair Th17 cell differentiation and alleviate MASH development (Ren et al., 2025).

NK cells may play a key role in maintaining inflammatory balance by secreting IFN-γ, thereby helping preserve tissue structural stability and suppressing the progression of MASH to liver fibrosis. Specifically, IFN-γ derived from NK cells promotes macrophage polarization toward the M1 phenotype, which, in turn, limits fibrogenesis. Conversely, depletion of NK cells triggers hepatocyte death and drives macrophages toward the M2 phenotype, causing TGF-β secretion and accelerated fibrosis (Notas et al., 2009; Tosello-Trampont et al., 2016). Consistent with this mechanism, studies have shown that NK cells can be activated at the MASH stage. However, research using Nfil3 knockout mice lacking NK cells reveals that MCD and CDHF diets-fed MASH is unexpectedly alleviated. Mechanistically, NK cells in MASH mice secrete a series of pro-inflammatory cytokines, including IFN-γ, IL-1β, IL-12, CCL4, CCL5, and granulocyte-macrophage colony-stimulating factor (G-MCSF), which can activate the JAK-STAT1/3 and NF-κB signaling pathways in the liver, thereby inducing hepatocyte apoptosis and promoting the progression of MASH (Wang F. et al., 2021).

LSECs, a type of non-parenchymal hepatic cells, are also critical in MASLD pathogenesis. Under physiological and quiescent states, LSECs maintain HSCs by secreting vascular endothelial growth factor (VEGF). However, under pathological conditions, damaged LSECs adopt a profibrotic phenotype and directly activate HSCs. Furthermore, during the progression of liver fibrosis, VEGF derived from LSECs can stimulate KCs to secrete pro-inflammatory factors such as IL-6, TNF-α, and TGF-β, thereby indirectly exacerbating the fibrotic process (Xu et al., 2017). In the murine MASH model, the Notch signaling pathway in LSECs is activated, and LSEC-specific Notch activation aggravates the disease by suppressing endothelial nitric oxide synthase (eNOS) transcription (Fang et al., 2022). Meanwhile, under lipotoxic conditions, the transcriptional level of vascular cell adhesion molecule 1 (VCAM-1) in LSECs is upregulated through the mitogen-activated protein kinase (MAPK) signaling pathway; anti-VCAM-1 treatment significantly ameliorates liver inflammation and fibrosis by reducing the infiltration of pro-inflammatory monocytes into the liver (Furuta et al., 2021).

Under normal physiological conditions, HSCs remain in a quiescent state, do not express alpha-smooth muscle actin (α-SMA), and show low levels of proliferation and collagen synthesis. However, upon inflammatory or mechanical injury to the liver, HSCs are activated and transition from a quiescent to an activated phenotype. This activation causes accumulation of extracellular matrix proteins, disrupting liver architecture and impairing hepatic function (Yan et al., 2025). Moreover, as described previously, HSCs function as central effectors of liver fibrosis by engaging in crosstalk and communication with hepatocytes, LSECs, macrophages, T cells, and other cell populations, thereby contributing to MASLD-associated fibrosis. Additionally, single-cell RNA-seq data from MASH liver models have shown that HSCs secrete various factors that primarily target endothelial and immune cells. For instance, ligands such as WNT4, NTN1, EFNB1, BMP5, GDF2, GDF10, and SEMA3C are largely restricted to endothelial cells, whereas CCL2, CCL11, CXCL10, CXCL12, CXCL16, connective tissue growth factor (CTGF), and growth arrest–specific 6 (GAS6) predominantly act on immune cells, including macrophages, dendritic cells (DCs), and T and B cells (Xiong et al., 2019). It remains under investigation whether HSCs secrete cytokines that crosstalk with other cells in MASLD progression.

#### 2.1.2 miRNA-mediated intrahepatic effects on MASLD

Within the liver microenvironment, diverse cell types communicate through miRNA to influence the progression of MASLD (Table 1). These miRNAs regulate the genes involved in lipid metabolism, inflammatory response, insulin resistance, liver fibrosis, and other processes in MASLD.

**TABLE 1:** Intrahepatic cell-derived miRNAs in MAFLD progression.

miRNA	Source cells	Target molecules	Disease effect	PMID
miR-192–5p ↑	Hepatocytes	*Rictor* *SCD1,YY1*	MASH ↑ MASLD ↓	31782176 29290651,38254634
miR-34a ↑	Hepatocytes	*SIRT1, ENO3, HNF4α, PPARα*	MASH ↑	22902550, 37960269, 26100857, 26330104
miR-34a-5p ↑	Unknown	*SIRT1, PPARα, GREM2*	Fibrosis ↑ MSLD/MASH ↑	39515682, 36541217, 37422021 40720724
miR-122 ↑	Hepatocytes	*Sirt1*	MASLD ↑	31195981
miR-122–5p ↓	KCs hepatocytes	*PKM2*	MASH ↑	35563621
miR-223 ↓	Hepatocytes macrophage	*Cxcl10, Taz*	MASH/Fibrosis/HCC ↑	30964207, 33236445
miR-103 ↑	Hepatocytes	*FASN,SCD1*	MASLD ↓	32035613
miR-103–3p ↑	Unknown	*HBP1,ACOX1*	MASLD ↑	35848933,35606603
miR-27a ↑	Hepatocytes	*FAS, SCD1* *PINK1*	MASLD ↓ Fibrosis ↑	29101357 34853724
miR-32–5p ↑	Hepatocytes	*INSIG1*	MASLD ↑	37451670
miR-363–3p ↑	Hepatocytes	*INSIG1*	MASLD ↑	39103107
miR-421 ↑	Hepatocytes	*SIRT3*	MASLD ↑	38764033
Let-7b-5p ↑	Hepatocytes	*Adrb3*	MASLD ↑	35018737
miR-128–3p ↑	Hepatocytes	*PPARγ*	Fibrosis ↑	26783552
miR-1297 ↑	Hepatocytes	*PTEN*	Fibrosis ↑	33911465
miR-107 ↑	Hepatocytes	*DKK1*	Fibrosis ↑	34234100
miR-132 ↑	Unknown	*Pten, Sirt1, FoxO3*	MASLD/MASH ↑	28381526
miR-182–5p ↑	Unknown	*Cyld, Foxo1*	MASLD/MASH/HCC ↑	37298191
miR-188 ↑	Unknown	*ATG12*	MASLD ↑	32252024
miR-26a-5p ↑	Unknown	​	MASLD ↑	39701472
miR-376b-5p ↑	Unknown	*FGFR1*	MASLD ↑	36057399
miR-28a-5p ↑	Unknown	*MARCH7*	MASLD ↑	37337032
miR-802 ↑	Unknown	*AMPK*	MASH ↑	36126896
miR-690 ↑	KCs	*NADK*	MASH/Fibrosis ↓	35700738
miR-4524a-5p ↓	Unknown	*TBP*	MASH/Fibrosis ↑	40251185
miR-411–5p↑	M2 macrophage unknown	*CAMSAP1* *EIF4G2*	MASH ↑ MASLD ↓	35789846 39261317
miR-142–3p ↓	PBMC	*ACSL4*	MASH ↑	39291441
miR-142–5p ↑	Unknown	*NRG1*	MASLD ↑	39259911
miR-204–3p ↓	Macrophage	*ULK1*	MASLD/MASH/fibrosis↑	38331323
miR-33 ↑	Hepatocytes	*PGC1α,AMPKα*	MASLD/MASH/fibrosis↑	39190492
miR-93 ↑	Unknown	*SIRT1*	MASLD ↑	40228656
miR-21–5p ↑	Unknown	​	MASLD/MASH/HCC↑	37846804
miR-326 ↑	Unknown	*FGF1*	MASLD ↑	41513602
miR-320 ↓	Hepatocytes	*RFX1*	MASH ↑	40893671
miR-149–5p ↑	Unknown	​	MASLD ↑	39263327
miR-511–3p ↓	Hepatocytes	*Rock2*	MASLD ↑	39710000
miR-155–5p ↑	Unknown	*Socs1*	MASH/fibrosis ↑	40360368
miR-18a-5p ↓	Unknown	​	MASLD ↑	40980810
miR-141 ↑	Hepatocytes	*Sirt-1*	MASLD/MASH ↑	39631458
miR-328–3p ↑	Hepatocytes	*PPP2R5D*	MASLD ↑	41255311

##### 2.1.2.1 miR-192–5p

Serum levels of miR-192–5p in patients with MASLD have been shown to correlate positively with liver inflammatory scores and disease severity. Furthermore, hepatocytes have been shown to secrete exosomes containing miR-192–5p in both patients with MASH and high-fat high-carbohydrate (HFHC) diet-induced rat MASLD models. Lipotoxic hepatocyte-derived exosomal miR-192–5p activates CD11b^+^CD86^+^ pro-inflammatory M1 macrophages, promoting the release of inflammatory factors such as inducible nitric oxide synthase (iNOS), IL-6, and TNF-α. Mechanistically, miR-192–5p targets Rictor and modulates the Rictor/protein kinase B (Akt)/FOXO1 signaling pathway to induce macrophage activation, and then promotes MASLD progression (Liu X. L. et al., 2020). However, at the early stage of MASLD, namely MAFL, miR-192–5p inhibits the expression of fatty acid synthase (*FASN*) by targeting stearoyl-CoA desaturase 1 (*SCD-1*) and Yin Yang 1 (*YY1*), thereby reducing triglyceride accumulation (Liu et al., 2017; Ma et al., 2023). These findings indicate that the role of miR-192–5p in different stages of MASLD is inconsistent, which may be related to the different targets of miR-192–5p. Notably, exosomal miR-192–5p secreted by lipotoxic hepatocytes promotes inflammation, whereas miR-192–5p itself inhibits lipid accumulation within hepatocytes. This feedback regulation highlights the complicated functioning of miR-192–5p during MASLD progression.

##### 2.1.2.2 miR-34a and miR-34a-5p

miR-34a plays a multifaceted regulatory role in the progression of MASLD and affects various pathological processes, such as cell apoptosis, lipid metabolism, inflammatory responses, fibrosis, and insulin resistance, by regulating different targets. Clinical studies have shown that the expression level of miR-34a in the liver gradually increases from simple steatosis to MASH. Overexpression of miR-34a in hepatocytes can induce apoptosis, inflammation, and fibrosis, thereby accelerating the progression of MAFL to MASH (Xu et al., 2021). Mechanistically, miR-34a can target sirtuin 1 (*SIRT1*) and its downstream molecules to promote cell apoptosis, including p53, p66shc, and FXR, forming a SIRT1-mediated signaling network. Interestingly, administration of carnosic acid can significantly alleviate hepatic oxidative stress and fibrosis by inhibiting miR-34a, which is followed by activation of the SIRT1/p66shc pathway (Castro et al., 2013; Alshehri et al., 2021).

miR-34a has been also reported to participate in glucose and lipid metabolism disorder. For instance, targeting enolase 3 (*ENO3*) by miR-34a can inhibit insulin signal transduction in hepatocytes and impair glucose metabolism; targeting hepatocyte nuclear factor 4α (*HNF4α*) can promote triglyceride accumulation; and targeting *SIRT1* and *PPARα* can weaken adenosine monophosphate (AMP)-activated protein kinase (AMPK) signaling and block fatty acid oxidation (FAO). Concurrently, miR-34a activates sterol regulatory element binding protein 1c (I) to enhance DNL. Finally, these effects exacerbate lipid deposition in hepatocytes and accelerate the progression of MASLD (Ding et al., 2015; Xu et al., 2015; Wang Y. et al., 2023). These findings demonstrated that the expression level of miR-34a-5p is positively correlated with body weight, liver function, and alanine aminotransferase (ALT) levels in MASLD mice. Notably, Artemisia and artemether can activate *SIRT1* and *PPARα*, thereby decelerating the progression of MASLD (Chen et al., 2022; Liang et al., 2025). In addition, miR-34a-5p has been shown to exacerbate MASLD-associated liver fibrosis by targeting *Grem2*, which activates the TGF-β signaling pathway to induce HSC activation (Shen et al., 2023). Furthermore, in patients with MASLD, a cholestatic profile is associated with worse outcomes. Deficiency of the transcription factor E2F2 can prevent MASH and cholestasis by modulating miR-34a-5p, thereby inhibiting cholesterol accumulation, fibrosis, and inflammation (Apodaka-Biguri et al., 2025).

##### 2.1.2.3 miR-122 and miR-122–5p

Recent studies have demonstrated that serum levels of circulating miR-122 fluctuate in accordance with the pathological changes associated with HCC and MASH. miR-122 expression shows a decreasing trend before the progression of fibrosis. Furthermore, some studies have shown that low expression of miR-122 in HCC tissue is significantly associated with poor overall survival in patients undergoing radical resection. However, circulating miR-122 levels do not predict overall survival, indicating that miRNA expression in tissue and blood are not always correlated (Akuta et al., 2019). In a murine MASLD model fed a high-fat diet (HFD) for 8 weeks, upregulation of miR-122 in hepatocytes was shown to target *Sirt1* and further promote the expression of lipogenic genes such as *SREBP1, FASN, SCD1, ACC1*, and apolipoprotein A5 (*ApoA5*) (Long et al., 2019). Additionally, in the KCs of MASH mice, downregulation of miR-122–5p has been shown to enhance the expression of pyruvate kinase muscle isozyme 2 (*PKM2*), thereby accelerating glycolysis in KCs (Inomata et al., 2022). Moreover, miR-122–5p from cholesterol-damaged hepatocytes activates M1 macrophages and exacerbates tissue inflammation during the progression of MASLD (Zhao et al., 2020b).

##### 2.1.2.4 miR-223

miR-223, a well-established anti-inflammatory miRNA, is highly expressed in the serum of patients with MASLD, and its expression shows a positive correlation with disease progression. miR-223 has been demonstrated to be expressed in hepatocytes. Moreover, miR-223-knockout mice exhibit more severe MASH phenotypes, which are characterized by elevated levels of inflammatory factors, exacerbated steatosis, upregulation of adipogenic genes, and increased fibrosis. miR-223 also targets inflammatory and oncogenic genes such as *CXCL10* and *Taz*, thereby suppressing the progression from fatty liver to MASH, fibrosis, and HCC (He et al., 2019). The elevation of miR-223 levels in hepatocytes may be derived from neutrophils and macrophages. Fatty acids stimulate neutrophils to secrete apolipoprotein E (ApoE)-enriched extracellular vehicles (EVs) binding with the higher expression of the low-density lipoprotein receptor (LDLR) in hepatocytes, further enhancing their ability to uptake miR-223-enriched EVs (He et al., 2021). Additionally, IL-6 promotes the release of miR-223-enriched exosomes from macrophages or neutrophils. These exosomes containing miR-223 migrate to hepatocytes and inhibit the expression of profibrotic genes, including *Taz*, *Nlrp3, Cxcl10,* and *Igf1r*, thereby suppressing MASLD-associated liver fibrosis. Interestingly, in PA-treated myeloid-specific IL-6ra KO mice, IL-6 promoted exosome secretion from macrophages and neutrophils, while miR-223 was shown to be released from macrophages-derived exosome, but not from neutrophils (Hou et al., 2021).

##### 2.1.2.5 miR-103 and miR-103–3p

miR-103 exhibits diverse biological functions, including suppression of tumor angiogenesis and tumor cell proliferation as well as regulation of adipogenesis and insulin sensitivity. Increased expression of miR-103 has been detected in the serum of patients with MASLD, while it has been viewed as a risk factor for insulin resistance (Xu, 2015). In sodium oleate-treated hepatocytes, miR-103 reduces lipid accumulation by directly targeting *FASN* and *SCD1*. Adenovirus-mediated overexpression of miR-103 in the liver of mice has been shown to inhibit DNL, thereby ameliorating hepatic steatosis in both HCD-fed and genetically engineered obese mice (Zhang et al., 2020). Furthermore, the expression of miR-103–5p is significantly upregulated in the serum and liver tissues of patients with MASLD. Moreover, miR-103–5p promotes lipid accumulation, hepatic inflammation, and liver fibrosis by targeting high-mobility group box transcription factor 1 (*HBP1*) and acyl-CoA oxidase 1 (*ACOX1*) (Chu and Gu, 2022; Ding et al., 2022).

##### 2.1.2.6 miR-27a

miR-27a has been reported to inhibit hepatic DNL by directly targeting *FASN* and *SCD1* in the liver, thereby alleviating obesity-induced MASLD (Zhang et al., 2017). Additionally, serum exosomal miR-27a levels are positively correlated with the severity of liver fibrosis, indicating that miR-27a could be a potential diagnostic marker and therapeutic target for liver fibrosis. Notably, FFAs can stimulate hepatocytes to release exosomal miR-27a, which negatively regulates *PINK1*, impairs mitophagy, and activates HSCs to induce MASLD-related fibrosis (Luo et al., 2021b). These data indicate that miRNA can play distinct roles at different stages of MASLD.

##### 2.1.2.7 miR-32–5p, miR-363–3p, miR-421 and let-7b-5p

Resource analysis revealed that hepatocyte-derived miRNAs are key regulators during the pathogenesis of MASLD. In both *in vivo* and *in vitro* MASLD models, miR-32–5p was highly expressed in hepatocytes, which can activate the SREBP1-mediated lipogenic program and promote hepatic lipid accumulation and metabolic dysregulation by directly binding to insulin-induced gene 1 (*INSIG1*). Hepatocyte-specific deletion of miR-32–5p ameliorates HFD-induced hepatic steatosis (Wang Y. D. et al., 2023). Correspondingly, hepatocyte-specific knockout of *INSIG1* promotes the expression of nuclear translocation of SREBP1 and activates downstream lipogenic gene expression leading to lipid accumulation. Furthermore, in both liver tissues of HFHC diet-fed mice and fatty acid-induced hepatocytes, miR-363–3p expression was significantly upregulated and then directly suppressed *INSIG1*, facilitating SREBP cleavage and nuclear translocation to activate lipogenic gene transcription (Wang L. et al., 2024).

MASLD is also a major complication of obstructive sleep apnea (OSA). In patients with OSA, hepatocytes secrete exosomal miR-421, which can target the sirtuin 3 (SIRT3)/AMPK pathway in macrophages to induce M1 macrophage polarization, thereby inhibiting autophagy and promoting NLRP3 inflammasome activation to accelerate MASLD progression (Yang et al., 2024). Notably, both palmitic acid (PA) and TGF-β stimulate hepatocytes to secrete let-7b-5p, which subsequently targets Adrb3 in adipocytes via the bloodstream. Consequently, let-7b-5p suppresses mitochondrial oxidative phosphorylation and impedes the browning of white adipose tissue, thereby exacerbating obesity and MASLD (Zhao et al., 2022). Except that, hepatocytes also secrete more miRNAs, such as miR-128–3p, miR-1297, and miR-107, which can target *PINK1*, *PPARγ*, *PTEN*, and *DKK1* respectively, leading to HSCs activation and MASLD-associated fibrosis (Povero et al., 2015; Luo et al., 2021a; Wang W. et al., 2021).

##### 2.1.2.8 miR-132 and miR-182–5p

Several miRNAs exhibit consistent regulatory effects throughout each pathological stage during the progression of MASLD. These miRNAs may better facilitate the diagnosis of patients with MASLD but cannot indicate the specific disease stage. To validate the clinical findings in patients with MASLD, researchers employed MASLD and MASH mouse models as well as transgenic mice overexpressing miR-132 and revealed that continuous hepatic overexpression of miR-132 can reduce the expression of its target genes, including *FoxO3*, *Pten*, and *Sirt1*. However, knockout of *FoxO3*, *Pten*, or *Sirt1* all failed to fully recapitulate the fatty liver phenotype induced by miR-132 overexpression. Notably, concurrent inhibition of *FoxO3*, *Pten*, and *Sirt1* produced cumulative effects in mice with diet-induced obesity, resulting in low-density lipoprotein (LDL)/vLDL levels similar to those in miR-132-overexpressing mice (Hanin et al., 2018).

miR-182–5p overexpression in the livers of MASLD mice as well as MASLD-related tumors mediated downregulation of the tumor suppressor genes *CYLD* and *FOXO1*. However, unexpected fluctuations in miR-182–5p overexpression were observed in mice fed a normal diet after 18 months, which could be attributed to progressive liver injury or the effects of aging (Compagnoni et al., 2023). MASLD predominantly affects middle-aged and older adults, since the associated risk factors typically escalate with advancing age (He et al., 2023).

##### 2.1.2.9 miR-188, miR-28a-5p, miR-376b-5p, miR-26a-5p and miR-802

In mice fed an HFD, the expression of hepatic miR-188, miR-28a-5p, and miR-376b-3p was significantly upregulated, exacerbating liver steatosis. Among these, miR-188 was identified as a negative regulator of hepatic glucose and lipid metabolism. Because autophagy plays a critical role in glucose and lipid metabolism, miR-188 promotes triglyceride accumulation in the liver and attenuates fatty acid β-oxidation, reducing hepatic insulin sensitivity and disrupting glucose homeostasis (Qian et al., 2021). Subsequent studies revealed that miR-188 regulates the autophagy process by targeting the autophagy-related gene 12 (*Atg12*), thereby influencing hepatic glucose and lipid metabolism and the progression of steatosis (Liu Y. et al., 2020).

miR-26a-5p promotes the protein kinase C delta (PKCδ) and TLR4/NF-κB signaling pathways and simultaneously inhibits the expression of the key autophagy protein Beclin 1, exacerbating hepatic inflammation, oxidative stress, and apoptotic damage (Basha et al., 2025). miR-376b-3p regulates lipid oxidation by targeting fibroblast growth factor receptor 1 (*FGFR1*), further influencing hepatic lipid accumulation (Wang X. Y. et al., 2022). In addition, miR-28a-5p is highly expressed in *ob/ob* mice and patients with MASLD, which downregulates the expression of MARCH7. As a ubiquitin E3 ligase, MARCH7 inactivation fails to induce the degradation of NLRP3 protein, thereby aggravating the progression of MASLD (Chen et al., 2023). Furthermore, obesity-induced expression of miR-802 can directly target AMPK and promote MASH in mice.

##### 2.1.2.10 miR-690, miR-4524a-5p, miR-411–5p, and miR-204–3p

Hepatic macrophages play pivotal roles in maintaining liver homeostasis and are critically involved in both injury and repair associated with acute and chronic liver diseases. KCs release exosomal miR-690, which acts on hepatocytes and HSCs to suppress liver fibrosis, DNL, and inflammatory responses. However, the expression of miR-690 in KCs is significantly reduced during MASH development, which subsequently affects its levels in HSCs and hepatocytes and thereby promotes the progression of MASH. *Nadk* is a target gene of miR-690. At the MASH stage, the expression level of NADK increases, while the level of NAD^+^ decreases. Supplementation with NAD^+^ can yield effects similar to those of miR-690 treatment (Gao et al., 2022). Furthermore, studies have shown that *Tim3* expression is elevated in hepatic macrophages in a murine fibrosis-associated MASH model induced by an MCD diet or HFD. In comparison with mice fed a normal chow diet (NCD), MCD diet exhibited lower levels of miR-4524a-5p in the liver. In MASH-related liver fibrosis, decreased expression of miR-4524a-5p weakens its interaction with TATA-binding protein (TBP), leading to upregulation of *TIM3* expression, which in turn induces M2 macrophage polarization and accelerates the process of liver fibrosis (Li et al., 2025). Additionally, M2 macrophage-derived exosomal miR-411–5p has been shown to impede the activation of HSCs by targeting calmodulin-regulated spectrin-assisted protein 1 (*CAMSAP1*) in a MASH model (Wan et al., 2022). Moreover, upregulation of miR-411–5p inhibited EIF4G2 to reduce FOXO3 expression, thereby reducing fatty acid synthesis and alleviating abnormal lipid deposition in MASLD (Wan et al., 2024). Emerging evidence suggests that miRNAs are also present and perform regulatory roles in the nucleus. Nuclear miR-204–3p levels are significantly reduced in hepatic macrophages, and the miRNA serves as a suppressor of MASH. Mechanistically, miR-204–3p alleviates MASH and liver fibrosis by orchestrating the effects of macrophages on hepatocytes and HSCs. The study demonstrated a newly identified effect of macrophage miR-204–3p, wherein it transcriptionally regulated ULK1 expression, thereby enhancing autophagy flux and attenuating inflammatory responses and ultimately limiting the progression of steatohepatitis (Zou et al., 2024).

##### 2.1.2.11 miR-142–3p and miR-142–5p

The expression of miR-142–3p has been shown to be reduced in the peripheral blood mononuclear cells (PBMCs) of children with MASLD, which may be associated with serum triglyceride levels (Oses et al., 2022). Metabolic diseases in adults, such as MASLD, may originate from the fetal period. Thus, paternal unhealthy lifestyle habits are a significant independent risk factors for developmental abnormalities and diseases in offspring. Pre-pregnant caffeine exposure (PPCE) has been shown to induce MASH in adult male offspring rats, resulting from paternal high glucocorticoid programming-induced hypermethylation of sperm miR-142–3p. Deficiency of miR-142–3p exacerbates PPCE-induced MASH in male offspring, while overexpression of miR-142–3p reverses the development of MASH. The targets of miR-142–3p include acyl-CoA synthetase, long-chain family member 4 (*ACSL4*), which promotes alterations in hepatic lipid metabolism in PPCE male offspring (Zhang et al., 2024). In addition, the study demonstrated that overexpression of circRRM2 ameliorates MASLD development by sponging miR-142–5p and upregulating neuregulin-1(NRG1) (Wu et al., 2024).

Except above mentioned miRNAs, other miRNAs involved in MASLD, such as miR-33, miR-93, miR-21–5p, miR-326, miR-320, miR-149–5p, miR-511–3p, miR-155–5p, miR-18a-5p, miR-141, miR-328–3p and their roles in the disease but not revealed completely are described in Table 1.

Thus, these studies demonstrate that the progression of MASLD is regulated by a complex regulatory network of cytokines and miRNAs, indicating that miRNAs hold substantial value in translational medicine. On one hand, the miRNA expression profiles involved in MASLD may act as diagnostic markers for disease progression. On the other hand, on the basis of the regulatory axis of “cytokine-miRNA-downstream target” in MASLD, new multi-target combination therapies can be designed.

### 2.2 The role of extrahepatic effects on MASLD

**FIGURE 1:**
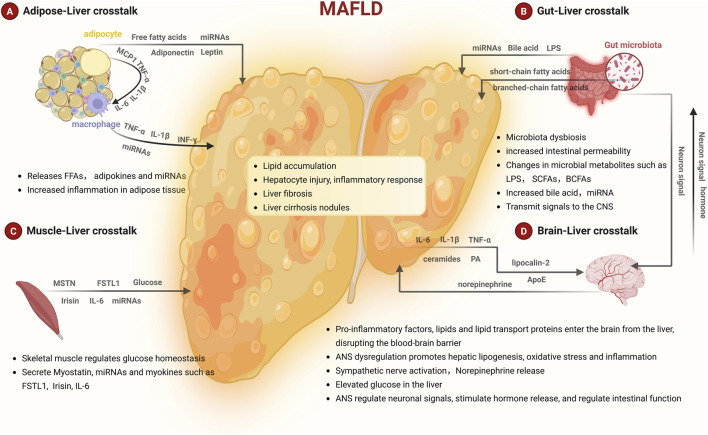
**(A)** Obesity induces adipocyte hypertrophy and rupture, leading to the release of FFAs into the liver, where they promote lipid accumulation. Additionally, adipocytes secrete adipokines and other mediators to attract macrophage recruitment, resulting in adipose tissue inflammation. ATMs release specific cytokines that directly promote lipolysis and increase lipid flux to the liver. **(B,D)** During MASLD progression, gut microbial dysbiosis enhances intestinal permeability, allowing substances such as LPS, bile acids, SCFAs, BCFAs, and miRNAs to flow into the liver and exacerbate the disease. Furthermore, the gut communicates with the CNS via the ANS, which in turn modulates intestinal function through neuronal signaling and hormonal release. In advanced MASLD stages, the liver releases pro-inflammatory factors, lipids, and lipid transport proteins into the circulation. These circulating factors will impair blood-brain barrier integrity and contribute to neurological pathology. Sympathetic overactivation stimulates hepatic glycogenolysis and gluconeogenesis, worsening hyperglycemia, and also acts directly on hepatic immune cells via norepinephrine release, thereby aggravating liver injury. **(C)** Skeletal muscle plays a critical role in regulating glucose homeostasis and lipid metabolism. It also secretes various myokines and factors, such as MSTN, miRNAs, FSTL1, Irisin, and IL-6, participate in the progression of MASLD.

**FIGURE 2:**
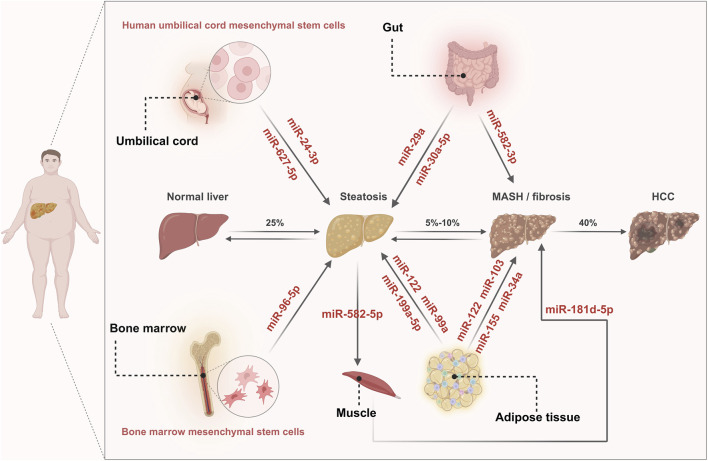
miRNA-mediated organ-organ communication. During the progression of MASLD, miR-24–3p and miR-627–5p derived from UC-MSCs, miR-96–5p derived from BM-MSCs, miR-199a-5p, miR-99a and miR-122 derived from adipose tissue, miR-29a and miR-30a-5p derived from the gut, and miR-582–5p derived from muscle, all participate in modulating liver steatosis. Additionally, miR-103, miR-122, miR-155 and miR-34a from adipose tissue, miR-582–3p from the gut, and miR-181d-5p derived muscle, exert regulatory effects on MASH/fibrosis.

The progression of MASLD involves multiple organ communication systems, including adipose-liver axis crosstalk, gut-liver axis crosstalk, muscle-liver axis crosstalk, as well as brain-liver axis crosstalk. These extrahepatic organs can secrete adipokines, cytokines, cellular metabolites (Figure 1), and even miRNAs to participate in MASLD progression. To date, miRNAs involved in extrahepatic organs have been shown to affect intrahepatic cells, including miR-122, miR-199a-5p, miR-103, and miR-99a from adipose tissue; miR-29a, miR-30a-5p, and miR-528–3p from the intestine; miR-582–5p and miR-181d-5p from muscle; miR-24–3p and miR-627–5p from mesenchymal stem cells (MSCs); as well as miR-96–5p from bone marrow (Figure 2).

#### 2.2.1 Adipose-liver communication in MASLD

##### 2.2.1.1 Inflammatory and adipokine mediate the crosstalk between adipose and liver during MASLD progression

Obesity and adipose tissue dysfunction are hallmarks of MASLD progression. Ectopic fat deposition that is a common feature of both obesity and MASLD may play a distinct role in liver diseases, and mediate fat-liver crosstalk via the portal vein (Gilani et al., 2024). The chronic metabolic stress associated with obesity promotes adipose tissue dysfunction and inflammation, resulting in the release of adipokines and a subsequent increase in hepatic lipid flux (Azzu et al., 2020). Adipose tissue, as the body’s the largest fat depot, stores excess calories and releases lipids when the body requires them. During the development of obesity, adipocytes undergo both hypertrophy and hyperplasia to accommodate energy storage in the form of triglycerides. However, the expansion capacity of adipocytes is limited. When over expanded, adipose tissue develops inflammation and dysfunction, promoting ectopic fat deposition in other depots, such as visceral and pericardial adipose as well as in organs including the liver, heart, and skeletal muscle.

In addition to serving as a lipid storage site, adipose tissue also releases FFA, which travel through the portal vein to the liver and contribute significantly to intrahepatic lipid accumulation (Hammarstedt et al., 2018; Hagberg and Spalding, 2023). In individuals with obesity, hypertrophied adipocytes secrete pro-inflammatory adipokines, such as monocyte chemoattractant protein-1 (MCP-1), TNF-α, IL-1β, and IL-6, which recruit substantial inflammatory monocyte infiltration and promote macrophage differentiation. These immune cells can account for up to 50% of the cells present in adipose tissue (Jorge et al., 2018; Zhu et al., 2024). Notably, the expression levels of IL-6 and TNF-α are elevated in the adipose tissue of obese patients with MASLD. Differentiated macrophages, namely adipose tissue macrophages (ATMs), release specific cytokines (e.g., TNF-α, IFN-γ, and IL-1β) that directly enhance adipose tissue lipolysis, increasing lipid flux to the liver (Daemen and Schilling, 2020). A growing body of evidence indicates that adipose tissue inflammation is linked to MASH. For instance, in MASLD mice, inflammation develops earlier in the epididymal white adipose tissue (eWAT) than that in mesenteric (mWAT) or inguinal white adipose tissue (iWAT), preceding the onset of overt MASH. Moreover, surgical removal of eWAT at the steatosis stage can reduce circulating inflammatory mediators, attenuate hepatic inflammation, and slow MASH progression (Mulder et al., 2015). In addition, transplantation of adipose tissue from obese but not lean mice increases hepatic macrophage infiltration and exacerbates MASH. Expectedly, deletion of ATMs before transplantation reverse these effects (Bijnen et al., 2018).

Leptin and adiponectin are key adipokines that also mediate the crosstalk between adipose tissue and the liver. Leptin, a peptide hormone encoded by the *Lepob* gene and secreted by adipocytes of white adipose tissue (WAT), regulates various physiological processes such as glucose metabolism and lipogenesis (Pereira et al., 2021). While leptin administration reverses hepatic steatosis in leptin-deficient *ob/ob* mice, supraphysiological levels promote hepatic inflammation and fibrosis (Perakakis et al., 2021). Consistently, in leptin-hyperresponsive MASH cirrhotic rats, leptin has been shown to induce HSC contraction, collagen deposition, and leukocyte recruitment (Yang et al., 2012). In addition to leptin, WAT secretes adiponectin, which also influences the progression of MASLD. Adiponectin, the most abundant adipose-specific hormone, acts primarily through adiponectin receptors 1 and 2 (AdipoR1 and AdipoR2). AdipoR1 is highly expressed in skeletal muscle and liver, while AdipoR2 is predominant in the liver. In the liver, AdipoR1 and AdipoR2 can activate the AMPK and PPARα pathways respectively, promoting glucose utilization and FAO (Li et al., 2022). During the progression of MASLD, adiponectin enhances the activity of carnitine palmitoyltransferase I (CPT1) to promote hepatic FAO, and it concurrently suppresses the activities of acetyl-CoA carboxylase (ACC) and fatty acid synthase (FASN) (Lee et al., 2023).

##### 2.2.1.2 miRNAs mediate the crosstalk between adipose and liver during MASLD progression

PTEN, which is derived from adipose tissue and functions as a key regulator of autophagy, is a target of miR-103. Autophagy plays a critical role in MASLD, governing essential hepatic functions such as glycogenolysis, gluconeogenesis, and β-oxidation. The selective degradation of damaged mitochondria through autophagy results in cellular injury and oxidative stress (Tsuji et al., 2023). Inhibition of miR-103 upregulates the expression of phosphorylated AMPKα (p-AMPKα), phosphorylated mTOR (p-mTOR), and p62, concurrently reduces lipid accumulation, and suppresses ALT/AST release in MASH further mitigating oxidative stress (Lu et al., 2023).

Several studies have demonstrated that miR-122 expression in the omental adipose tissue of obese patients is twofold higher than that in healthy individuals (Heneghan et al., 2011). Baranova et al. revealed that during the early stages of MASLD, hepatic miR-122 levels decrease, whereas elevated miR-122 expression in adipose tissue may compensate for its deficiency in liver. Over time, the compensatory secretion of miR-122 by adipose tissue continues to weaken, causing a reduction in exogenous miR-122 delivery to the liver and thereby accelerating the progression of MASLD and liver fibrosis (Baranova et al., 2018). Expectedly, miR-122 deficiency promotes the progression of MASLD, and the administration of antagomiR-122 in mice significantly suppresses miR-122 expression in blood, liver, and adipose tissue. Activation of the RORA agonist RS-2982 enhances miR-122 promoter activity; therefore, RS-2982 exhibits anti-MASLD/MASH effects, including reduced triglyceride accumulation, suppressed hepatic inflammation, reversal of fibrosis, and improved insulin resistance (Chai et al., 2020).

Furthermore, brown adipose tissue transplantation has been shown to improve glucose and lipid metabolism in diabetic mice and elevate circulating miR-99a levels. Experimental data indicate that miR-99a targets *NOX4* to ameliorate oxidative stress in LO2 cells (Li P. et al., 2020). miR-199a-5p, which is highly expressed in murine adipose tissue, suppresses the expression of mammalian sterile 20-like kinase 1 (*MST1*), thereby accelerating the formation of hepatic lipid droplets and disrupting lipid metabolism. *MST1*, a key component of the Hippo signaling pathway, plays a crucial role in regulating cell proliferation, apoptosis, autophagy and immune responses, and in maintaining metabolic homeostasis in the liver. The overexpression of *MST1* can reverse the lipid accumulation induced by miR-199a-5p (Li Y. et al., 2020). Moreover, in patients with MASLD, the AT-derived exosome miR-548ag upregulates FASN expression by inhibiting DNMT3B, causing enhanced hepatic lipid accumulation (Chu et al., 2025). Additionally, recent studies have shown that MASH-ATM-EVs can activate HSCs and promote liver fibrosis in MASH. Elevated levels of miR-155 and miR-34a originated from ATM-EVs downregulate *PPARγ* in MASH, thereby enhancing the profibrotic effects of HSCs (Rohm et al., 2025).

#### 2.2.2 Gut-liver axis communication in MASLD

##### 2.2.2.1 Gut microbiota and the effects of its metabolites on MASLD

The gut-liver axis refers to the bidirectional communication between the liver and the gastrointestinal tract or the gut microbiota, which plays a central role in regulating both physiological and pathological states of MASLD. The gastrointestinal barrier, which consists of microbial, chemical, physical, and immune components, maintains gut homeostasis and protects the body from bacteria, toxic metabolites, and antigen attacks. Gut microbiota dysbiosis and alterations in metabolites compromise the integrity of the gut barrier, causing increased intestinal permeability and exacerbating liver injury (Fernandez-Cantos et al., 2021). Firmicutes and Bacteroidetes together account for over 90% of the total gut bacteria and play beneficial roles. In animal models, specific bacterial strains such as *Lactobacillus*, *Bifidobacterium*, *Akkermansia muciniphila*, and *Bacteroides uniformis* have been shown to have protective effects against MASLD (Kim et al., 2020; Zhao et al., 2020a; Lee et al., 2021; Song et al., 2023).

Dysbiosis of the intestinal microbiota alters microbial metabolites, including the levels of LPS, an endotoxin derived from the cell wall of Gram-negative bacteria that acts as a classical pathogen-associated molecular pattern (PAMP). As a key gut-derived signal molecule in gut-liver crosstalk, LPS activates the TLR4 signaling pathway, causing activation of NF-κB and the production of pro-inflammatory cytokines, thereby exacerbating MASH progression (Panyod et al., 2024). Additionally, bile acids synthesized in the liver are secreted into the gut and subsequently metabolized into secondary bile acids by the microbiota in the intestine. Both primary and secondary bile acids are involved in the enterohepatic circulation, whereby they are reabsorbed in the intestine and returned to the liver via the portal vein. Although the physiological levels of bile acids facilitate intestinal barrier function, elevated concentrations of bile acids can impair barrier integrity (Tilg et al., 2022). Mechanistically, increased bile acid concentrations can activate the nuclear receptor FXR that is expressed in hepatocytes and intestinal epithelial cells. FXR activation induces negative feedback regulation of bile acid synthesis, reduces hepatic triglyceride accumulation, and decreases intestinal absorption of dietary lipids (Clifford et al., 2021).

The high-fat, typical Western diets that are rich in saturated fatty acids have contributed to MASLD development. In addition to directly damaging the liver, such diets also alter the composition and functionality of the gut microbiota. Conversely, probiotics such as *Bifidobacterium* produce short-chain fatty acids (SCFAs) and indole derivatives, which can ameliorate MASLD through modulation of the gut-liver axis (Yoon et al., 2023). However, the roles of SCFAs are complex and occasionally contradictory. Acetate, propionate, and butyrate are the most abundant SCFAs, and they account for 90%–95% of the total SCFAs in the colon. Interestingly, some studies have indicated that the above-mentioned SCFAs can improve hepatic steatosis by activating adipose tissue. In contrast, patients with MASLD show decreased levels of branched-chain fatty acids (BCFAs), especially isobutyrate and isovalerate; these fatty acids may increase the risk of MASLD (Tsai et al., 2023).

##### 2.2.2.2 The effect of gut-derived miRNAs on MASLD

An HFD diet alters the composition and abundance of gut microbiota, which serves as a driver of miRNA dysfunction in liver diseases. The gut influences hepatic lipid metabolism by transporting gut-derived substances, including miRNAs carried by exosomes, into the liver through the portal vein (Zhang et al., 2022). Yang YL et al. showed that HFD-fed mice exhibit increased abundances of Parabacteroides, *Bacteroides*, and Mucispirillum, which are associated with heightened inflammation. Notably, miR-29a modulates the expression of IL-6 and further reduces tissue inflammation, MASLD, as well as intestinal dysfunction in HFD-fed mice (Yang et al., 2023). Additionally, miR-30a-5p regulates gut microbiota through *ALOX5, ALOX12,* and *COX2*, which encode key enzymes in the arachidonic acid pathway, exacerbating hepatic steatosis in HFD-fed mice (Wang R. et al., 2024). Furthermore, several studies have revealed a correlation between miR-582–3p expression and fecal microbiota in MASH. miR-582–3p upregulates the expression of α-smooth muscle actin (α-SMA), type I collagen α1 chain (COL1A1), and fibronectin 1 (FN1) by targeting *TMBIM1*, thereby promoting hepatic stellate cell proliferation and advancing MASH (Huang et al., 2023). Therefore, to investigate gut-liver crosstalk which mediated by gut microbiota and its metabolites, as well as miRNAs may provide potential therapeutic strategies for MASLD.

#### 2.2.3 Muscle-liver communication in MASLD

##### 2.2.3.1 Muscle cytokines mediate the crosstalk between muscle and liver during MASLD progression

Skeletal muscle is essential for regulation of glucose homeostasis and lipid metabolism. The onset of insulin resistance in skeletal muscle disrupts insulin signal transduction and reduces insulin-stimulated glucose uptake, causing hyperglycemia and hyperinsulinemia (Pyun et al., 2021). Concurrent disturbances in glucose metabolism enhance hepatic glucose production, providing a substrate for DNL. Insulin further promotes fat accumulation and accelerates the progression of MASLD through activation of SREBP1c. Notably, muscle insulin resistance is characterized by impaired glucose utilization and elevated levels of non-esterified fatty acids in patients with MASLD (Marjot et al., 2025). Moreover, sarcopenia is strongly associated with MASLD, and detrimental changes in muscle composition are frequently observed in these patients (Linge et al., 2021). Myostatin (MSTN), a cytokine secreted by skeletal muscle and adipocytes, serves as a negative regulator of muscle protein synthesis, mainly through inhibition of rapamycin (mTOR) signaling pathway. Elevated serum myostatin (MSTN) levels, which are frequently observed in patients with liver cirrhosis and concomitant sarcopenia, are associated with a poor prognosis Similarly, in patients with MASLD-related HCC without advanced liver fibrosis, higher MSTN levels in serum are correlated with reduced overall survival (Yoshio et al., 2021). Additionally, MSTN can activate primary fibroblasts and promotes collagen production resulting in liver fibrosis.

Myokines, which are bioactive factors secreted by skeletal muscle, also contribute to the pathogenesis of hepatic steatosis, fibrosis, and cancer. For example, follistatin-like protein 1 (FSTL1), a secretory glycoprotein mainly produced by skeletal muscle, is a key myokine. In the HFD-induced MASH mouse model, serum FSTL1 levels showed a positive correlation with the severity of liver pathology, including steatosis, ballooning degeneration, fibrosis, and the MASLD activity score. Mechanistically, muscle-derived FSTL1 promotes the progression of MASLD through interferon regulatory factor 4 (IRF4)-mediated pathways (Guo et al., 2023). Irisin, which is cleaved from fibronectin type III domain-containing protein 5 (FNDC5), is released primarily by skeletal muscle as well as by liver and adipose tissue. Plasma irisin levels are decreased in HFD-induced MASLD mice, and exogenous irisin supplementation replenishes its concentration (Li et al., 2021). Another myokine, IL-6, is released during muscle contraction. Importantly, muscle-derived IL-6 exerts anti-inflammatory effects rather than pro-inflammatory actions. It modulates lipolysis and glyceroneogenesis in adipose and hepatic tissues. IL-6 knockout (KO) mice fed an HFD exhibited a reduction in the mass of both inguinal and epididymal WAT relative to body weight. Furthermore, caffeine has been shown to stimulate IL-6 secretion from skeletal muscle. Correspondingly, in HFD-induced MASLD mice, caffeine treatment was shown to increase plasma IL-6 levels, reduce triglyceride levels, and alleviate MASLD (Nara and Watanabe, 2021).

##### 2.2.3.2 miRNA-mediated muscle-liver crosstalk during MASLD progression

MASLD shows a well-documented correlation with sarcopenia, wherein the liver-muscle axis acts as a key mediator underlying this association. Using HFD-fed mice, one study demonstrated that hepatic injury can induce systemic inflammation and lipid disorders, which subsequently impair skeletal muscle and promote muscle atrophy. In addition, administration of reactive oxygen species (ROS)-sensitive hydrogels into MASLD mice showed that a reduction in the release of ROS can facilitate alleviation of liver inflammation and stop fibrosis. Meanwhile, ROS-sensitive hydrogel treatment also upregulated miR-582–5p in liver-derived exosomes and then downregulated the expression of the muscle atrophy-related genes *Fbxo32* and *Trim63*. *In vitro* experiments further confirmed that exosomal miR-582–5p derived from HepG2 cells can be delivered into C2C12 myoblasts, which consequently reduce the activation of atrophy-related pathways and preserve the integrity of myotubes (Jiang et al., 2025). The findings obtained by Zhao YC et al. further support the bidirectional communication along the liver-muscle axis during MASLD progression. Their results demonstrated that remote limb ischemic conditioning (RIC), a non-invasive muscle therapy, attenuates the MASH phenotype in mice. Specifically, miR-181d-5p derived from the muscle tissue of RIC-treated MASH mice reduces hepatocyte death and inflammation induced by PA while enhancing FAO. Furthermore, hepatocyte-specific overexpression of miR-181d-5p by tail vein injection of serotype 8 adeno-associated virus (AAV8) driven by the liver-specific thyroxine-binding globulin promoter has been shown to significantly alleviate hepatic steatosis, immune cell infiltration and fibrosis in MASH mice. The mechanisms underlying these therapeutic effects involve targeting of NR4A3 in hepatocytes through miR-181d-5p (Zhao et al., 2025).

#### 2.2.4 Brain-liver axis communication in MASLD

MASLD is associated with neuroinflammation and may elevate the risk of Alzheimer’s disease and Parkinson disease (PD). During the progression of MASLD, hepatic fat accumulation and metabolic dysregulation cause increased systemic levels of pro-inflammatory factors (IL-1β, IL-6, and TNF-α), lipids (ceramides and PA), and lipid transporters (lipocalin-2 and ApoE). These circulating mediators can enter the brain through the bloodstream by compromising the integrity of the blood–brain barrier and initiate neuropathological changes. Additionally, peripheral insulin resistance can contribute to systemic inflammation, which may promote cerebral insulin resistance and exacerbate neuroinflammation (Asimakidou et al., 2025). MASLD has also associated with cognitive impairment, brain dysfunction, behavioral changes, and total brain volume reduction. For example, MASLD mice with knockout of monocarboxylic acid transporter 1 (MCT1), a key transporter for SCFAs, ketone bodies, and lactate, showed cerebral hypoxia as well as morphological and metabolic alterations in microglia and astrocytes, respectively. No similar effects were observed in wild-type MASLD mice (Hadjihambi et al., 2023). The cognitive deficits associated with MASLD may also arise from disrupted neural activity and functional connectivity. In patients with pre-cirrhotic MASLD, reduced local coherence has been observed in the left parietal lobule, a region critical for attention and memory processing. Moreover, aberrant functional connectivity between the left parietal lobule and the posterior cingulate gyrus may further contribute to cognitive dysfunction (Shu et al., 2023). Dysregulation of the autonomic nervous system (ANS) also plays an important role in MASLD by promoting hepatic lipogenesis, oxidative stress, and inflammation. Excessive sympathetic activation can stimulate hepatic glycogenolysis and gluconeogenesis and worsen hyperglycemia by directly affecting liver immune cells through norepinephrine release, thereby aggravating liver injury (Wan, 2025).

The emerging concept of the brain-gut-liver axis encompasses communication among multiple organs, including the small intestine, intestinal vagus nerve, hypothalamus, and hepatic vagus nerve. Afferent pathways within the gut convey signals to the central nervous system through the ANS, which, in turn, modulates intestinal function through neural signaling and hormonal release. Notably, various microbial metabolites, such as serotonin, dopamine, norepinephrine, SCFAs, glutamate, γ-aminobutyric acid (GABA), secondary bile acids, tryptophan metabolites, and histamine, are involved in these communication pathways. The release of these signaling molecules can convey physiological changes to both the brain and the liver (Chiang and Ferrell, 2019; Rebelos et al., 2021; De Cól et al., 2024).

Direct evidence indicating that the brain-liver axis communication is regulated by miRNAs is lacking. However, recent studies have shown that miR-31–5p in EVs derived from MSCs can promote the transition of liver macrophages from pro-inflammatory M1 to anti-inflammatory M2 phenotypes in MASH mice by regulating platelet-derived growth factor B (*PDGFB*). Notably, high concentrations of PDGFB are known to exacerbate pericyte apoptosis. Moreover, pericytes play a crucial role in maintaining blood–brain barrier integrity, regulating vascular remodeling, and supporting neurovascular integrity. After treatment with miR-31–5p agomiR, neurovascular integrity is restored in mice receiving an MCD diet (Du et al., 2025). These findings indicate that cross-organ intervention strategies targeting the brain-liver axis holds promise for treating MASLD-related metabolic abnormalities and neurological complications.

#### 2.2.5 The other forms of organ–organ communication in MASLD

Mesenchymal stem cells (MSCs) exhibit remarkable capabilities for multidirectional differentiation, self-renewal, immunomodulation, and long-term proliferation. MSCs, which play pivotal roles in tissue regeneration and the treatment of diseases, can be isolated from various sources, including bone marrow, adipose tissue, dental pulp, placenta, and umbilical cord (Patel et al., 2024). Human umbilical cord mesenchymal stem cell (hUC-MSC)-derived miR-24–3p specifically targets and suppresses Kelch-like ECH-associated protein 1 (Keap1). Keap1 facilitates the continuous ubiquitination and degradation of cytosolic nuclear factor erythroid 2-related factor 2 (Nrf2), which is crucial for protecting cells from ROS generation and preventing oxidative damage in hepatocytes. By targeting Keap1, miR-24–3p effectively reduces lipid accumulation, ROS production, and inflammatory responses, thereby alleviating the progression of MASLD (Du et al., 2022). Furthermore, Marwa O. El-Derany et al. also demonstrated that hUC-MSC-derived miR-627–5p inhibits the expression of the fat mass and obesity-associated gene (*FTO*). FTO is significantly overexpressed in MASLD animal models and promotes lipid accumulation in hepatocytes by accelerating the maturation of SREBP1c, which in turn enhances the expression of lipid droplet-associated proteins. miR-627–5p can downregulate the expression of Fas and SREBP1c while upregulating the lipid homeostasis regulator PPARα expression in PA-treated LO2 cells (Cheng et al., 2021).

Bone marrow mesenchymal stem cells (BM-MSCs), the most well-characterized and commonly utilized source of MSCs, can secrete miR-96–5p to downregulate fatty acid synthesis (*SREBP1/2, ACC*) and lipid uptake (*CD36*) by targeting caspase-2. Moreover, they can upregulate fatty acid oxidation-related molecules containing PPARα and CPT1 to mitigate the progression of MASLD/MASH (El-Derany and AbdelHamid, 2021). As stem cell therapies gain traction in clinical practice, certain limitations of allogeneic transplantation have become evident, including potential immune reactions and oncogenic risks. In contrast, miRNA-based therapeutics can circumvent these limitations and show substantial advantages for clinical applications.

In summary, these studies have characterized the pivotal role of cytokine- and miRNA-mediated multiorgan interactions during the progression of MASLD, providing a critical theoretical foundation for the pathogenesis and associated complications of MASLD. Importantly, future therapeutic strategies should account for multiorgan crosstalk during MASLD progression and then design more reasonable target interventions for MASLD treatment.

## 3 Target miRNAs for MASLD therapy

Although pharmacotherapy is currently the primary method for treating MASLD, pharmacotherapeutic agents may exhibit adverse effects. For instance, certain antifibrotic drugs can markedly suppress HSC activation while activating other cell types, complicating the reversal of fibrosis (Roehlen et al., 2020). miRNAs, which can target multiple genes, may simultaneously regulate compensatory pathways and offer unique therapeutic advantages as endogenous molecules (Su et al., 2018). Additionally, small nucleic acid drugs can be designed with sequences that specifically target genes, thereby avoiding indiscriminate development. This targeted approach can significantly accelerate the research and development process, reduce costs, and improve specificity.

miRNA therapeutics are categorized into two classes: (1) miRNA mimics, which are chemically synthesized endogenous miRNAs that enhance native miRNA functions, and (2) miRNA inhibitors, including ASOs, locked nucleic acid (LNA) anti-miRNAs, anti-miR oligonucleotides (AMOs), small molecule inhibitors of miRNAs (SMIRs), antagomiRs, miRNA-zippers, and miRNA sponges, which can disrupt miRNA-RNA interactions (Petrovic and Ergun, 2018). Several studies have shown that exosomal miR-690 derived from M2 bone marrow-derived macrophages (BMDMs) enhances insulin sensitivity (Ying et al., 2021). Moreover, miR-690 suppresses the NF-κB pathway to promote osteogenic differentiation (Yu et al., 2016). Using experiments conducted in high-sucrose and cholesterol diet-fed MASH mice, Hong et al. demonstrated that delivery of miR-690 mimic-loaded nanoparticles can attenuate hepatic fibrosis, steatosis, and inflammation. Cellular analyses have shown restored phagocytic function in KCs as well as decreased secretion of pro-fibrogenic factors and lipogenic mediators in hepatocytes (Gao et al., 2022). In MASH mice fed an HFHC diet for 16 weeks, injection of AAV-miR-145a-5p mimics through the tail vein markedly alleviated diet-induced liver injury, including inflammation and fibrosis, and simultaneously improved glucose metabolism and insulin sensitivity (Li et al., 2023). The effectiveness of miR-345–5p has been implicated in various pathologies, including alleviation of rhinitis by downregulating the TLR4/NF-κB pathway in nasal epithelial cells. Analyses from the Gene Expression Omnibus (GEO) database further indicated a reduction in miR-345–5p expression in cases of human hepatic fibrosis (Chen et al., 2017). Administration of miR-345–5p mimics through the tail vein has been shown to attenuate liver fibrosis in murine models induced by carbon tetrachloride (CCl4), bild-duct ligation (BDL), or an HFD (Wang P. et al., 2022). Although data regarding miRNA-based therapies for MASLD remain limited, the promising outcomes of miRNA mimics in preclinical MASLD models indicate their potential for future therapy applications.

Notably, no clinical trials investigating miRNA-targeted interventions for MASLD have been reported to date, reflecting the inherent challenges in the clinical translation of miRNA therapeutics. Miravirsen, a nucleic acid-modified DNA phosphorothioate ASO, suppresses miR-122 activity by stabilizing it within highly durable heteroduplex complexes. A phase IIa study published in *The New England Journal of Medicine* demonstrated long-term, dose-dependent reductions in HCV RNA levels among 36 HCV-infected patients who received Miravirsen injections (Janssen et al., 2013). MRX34 is a tumor-targeting drug based on a miR-34a mimic, but its clinical trial was terminated due to the appearance of immune-related adverse events in five cases (Zhang et al., 2019). Although Miravirsen has been developed for HCV infection treatment, emerging evidence suggests that miR-122 also plays regulatory roles in the pathogenesis of MASLD, implying its therapeutic potential. Nevertheless, the termination of the MRX34 trial highlights the need to rigorously balance therapeutic efficacy and immunogenic safety in future clinical translation efforts.

The existing miRNA delivery technologies predominantly employ synthetic materials such as lipid-based delivery systems, polyethyleneimine (PEI)-based systems, and poly (lactide-co-glycolide) (PLGA) particles as well as viral vectors. Synthetic delivery systems are becoming increasingly prominent in miRNA therapeutics due to their ease of production, operational versatility, high biocompatibility, and low immunogenicity. Advanced delivery platforms can be designed to protect nucleic acid therapeutics from endonuclease degradation *in vivo* while enhancing cellular uptake efficiency. MSCs, which are pluripotent stem cells with self-renewal capacity, are ubiquitously present in adult tissues. However, MSC transplantation is associated with major clinical challenges, including risks of immune rejection and tumorigenic potential (Zou et al., 2023). A growing body of evidence indicates that the therapeutic effects of stem cells may be attributable to exosomal miRNAs secreted by MSCs. Dong Jun Park et al. developed iron oxide-incorporated PLGA nanoparticles to augment exosome production from MSCs (Park et al., 2020). Biomaterial-derived non-viral vectors or biological carriers, such as exosomes, play pivotal roles in efficient miRNA delivery (Jo et al., 2019). Engineered exosomes miRNA may be utilized to treat MASLD by harnessing their natural homing capabilities.

Conventional delivery systems, including nanomaterials and viral vectors, often show suboptimal hepatic targeting. *N*-acetylgalactosamine (GalNAc) conjugation can serve as a ligand for the asialoglycoprotein receptor (ASGPR), a highly specific endocytic receptor that is predominantly expressed on hepatocyte membranes and shows minimal presence in other cell types (Haussecker, 2014). The GalNAc-coupled small-nucleotide delivery system achieved liver specificity. Given the large molecular weight and hydrophilic properties of siRNA, which hinder cellular penetration, GalNAc-based platforms represent an ideal strategy for siRNA delivery. Sorafenib, a first-line therapeutic agent for advanced HCC, has been shown to serve as a potent ferroptosis inducer. URB1-AS1 has been shown to attenuate Sorafenib-induced ferroptosis by promoting ferritin phase separation and reducing intracellular free iron levels. Notably, GalNAc-small interfering (si)URB1-AS1 suppresses URB1-AS1 expression in HCC, thereby enhancing tumor cell sensitivity to Sorafenib (Gao et al., 2023). These studies indicate the potential applicability of liver-specific miRNA delivery based on the GalNAc system in the future therapeutic context.

## 4 Conclusion and challenge

In the progression of MASLD, miRNA-mediated inter-organ communication offers considerable potential for diagnosing and staging the disease. Moreover, in comparison with conventional RNA therapies, miRNAs offer distinct advantages such as reduced immunogenicity, improved therapeutic precision, and enhanced structural stability. Notably, the current diagnostic gold standard for the diagnosis of MASLD is still liver biopsy, and the detection of non-invasive serum miRNA biomarkers has broad prospects.

However, the application of miRNAs in the diagnosis and treatment of MASLD is still associated with multiple challenges. First, a single miRNA often regulates multiple target cells and genes, and synergistic effects on these targets facilitate the treatment of MASLD. Conversely, inconsistent or opposing actions may yield complicated therapeutic outcomes. A notable example is miR-192–5p, which shows stage-specific behavior. At the early stage of MASLD, miR-192–5p targets SCD1 and YY1, thereby suppressing FASN expression, reducing triglyceride accumulation, and attenuating disease progression. In contrast, during MASH, lipotoxic hepatocytes secrete miR-192–5p to target Rictor, activating pro-inflammatory macrophages and exacerbating MASLD. This form of stage-related and functional heterogeneity hinders the diagnostic and therapeutic potential of miRNAs for MASLD. Second, MASLD is a metabolic syndrome encompassing a spectrum of liver diseases. Most of the existing studies have employed single models to study specific stages of MASLD, limiting the ability to precisely diagnose the development of MASLD stages using a single miRNA. Third, from a therapeutic perspective, miRNAs are naked RNA molecules that are highly susceptible to degradation by nucleases *in vivo*. Moreover, their negative charge and hydrophilic nature hinder efficient cellular membrane penetration. Recent studies have highlighted lipid nanoparticles (LNPs) as a promising delivery platform, offering multiple advantages such as effective RNA encapsulation, protection from enzymatic degradation, and enhanced cellular uptake through membrane fusion. However, their long-term efficacy and safety require evaluation. Furthermore, the tissue- and cellular-targeting specificity of LNPs require further optimization to achieve precise therapeutic delivery. Although existing LNP systems can achieve liver-targeted delivery, they lack the precision to target specific hepatic cell populations. In this regard, investigating the differences in target cells such as hepatocytes, HSCs, and other immune cells may help improve miRNA-targeting precision.

In response to these challenges, future studies should prioritize technological innovation, particularly leveraging artificial intelligence to design delivery vectors with improved targeting specificity. By integrating multi-omics analyses, a more comprehensive understanding of the role of miRNAs-mediated multiorgan communication networks in MASLD can be characterized. Furthermore, establishing clinical cohorts will be essential to validate the utility of miRNAs as biomarkers for MASLD diagnosis, thereby providing a solid foundation for personalized therapeutic strategies.
